# Progress in stratified stroke characterization—associated with better diagnosis, accurate prognosis and improved treatment strategies

**DOI:** 10.1111/bpa.13149

**Published:** 2023-01-13

**Authors:** Mark Slevin

**Affiliations:** ^1^ George Emil Palade University of Medicine, Pharmacy Science and Technology of Targu Mures Targu Mures Romania; ^2^ The School of Life Sciences Manchester Metropolitan University Manchester UK

**Keywords:** biomarkers, diagnosis, prognosis, stratified medicine, stroke

In this special issue, focussed on novel stroke biomarkers, we invited world‐leading researchers to submit novel findings from work originating from the Asian provinces of China. As we move towards an era of stratified and personalised medicine with a significant input from large data sets and artificial intelligence (AI) algorithms, a complete understanding of the clinical background, risk factors, genetics and epigenetic markers and pathophysiological biomarkers that inform and therefore predict response to treatment and ultimately, outcome, are still needed [1]. Therefore, here, are included several articles that provide further novel insight into this complex disease process, and which will contribute to the synthesis of data resulting in our transcendence to optimized treatment and rehabilitation with associated reduced morbidity and mortality.

The contribution of inflammation and the neuroinflammatory response to stroke volume, neuronal cell death and outcome is undeniable [2], with activation of local microglia stimulating and pre‐empting the arrival of an ‘army’ of inflammatory M2‐polarised monocytes/macrophages through the damaged blood–brain barrier secreting damaging cytokines such as interleukins 1‐β/6/8, as well as tumour necrosis factor‐α, and others including the more recently identified plasma‐borne monomeric C‐reactive protein [3, 4]. In this series, Zhang et al. [5], provided a comprehensive review of the role of the mononuclear phagocyte system in ischaemic stroke, summarizing that the effects are time dependent and influence the processes of stroke volume development, angiogenesis and remodelling and plasticity, as well as being potential markers of long‐term prognosis and outcome. Hence as recently described by Monsour et al. [6], targeting critical early inflammatory pathways such as pro‐inflammatory IL‐6 but protecting the inactive IL‐6‐soluble IL‐6 receptor 130 complex might form part of a future combinational therapeutic effort.

Within this series, and in an original and more translational study, Han‐Yu et al. [7], analysed the impact of reactive astrogliosis following transient global cerebral perfusion using a rat model of stroke. They showed that conditional knockout of the lysosomal‐associated membrane protein 2A (LAMP‐2A), protected CA1 hippocampal neurones from death also ameliorating spatial learning and memory deficits, by inhibiting astrocyte activation. They proposed this as a potential novel neuroprotective strategy. Whilst additional protective mechanisms include the prevention of apoptotic protein translocation (Bax/Bad), both the previous findings that LAMPs are involved in mediating proper autophagy that could protect the brain against toxic plaque build up‐also associated with the medium to long‐term consequences after stroke, as well as the known limitations in translation of rodent models to the human condition [8]. Nevertheless this work represents an important finding within the field.

As mentioned earlier, AI algorithms and deep‐learning predictive big‐data‐derived technology will be essential to improve our understanding of how to manage stroke at the individual level, for example, by providing MRI‐lesion segmentation datasets that will help in treatment decision‐making and evaluating outcome. In this series, Jiang et al. [9] showed that a multiparameter model using deep‐learning automated processes, measuring datasets of the volume of interest and segment slices, together, significantly better predicted haemorrhagic transformation in patients following endovascular thrombectomy after ischaemic stroke, compared with single parameter models. One of the less common causes of cerebral infarction is susceptibility due to vertebrobasilar dolichoectasia (VBD), where abnormalities in the basilar artery predispose to premature development of atherosclerosis. Mortality rates are high due to the non‐specific symptoms and delay in diagnosis. Here, Tao et al. (2023), used high‐resolution MRI (HR‐MRI), to fully characterise the arterial structure and function in 24 patients and concluded that risk of stroke was associated with bifurcation height and arterial turbulence and inferior diameters [10]. This work should help to enable more effective stratified risk assessment following larger‐scale clinical studies.

In conclusion, this special series of articles reflects the broad and extremely complex nature of stroke pathophysiology. Importantly, it indicates how a series of small advances could eventually support an optimised programme of stratification and personalised approach to understanding risk, diagnosis prognosis and outcome. This would probably be achieved using a combination of data‐driven deep learning AI together with point‐of‐care rapid testing, through advanced imaging and likely a combinational approach to targeting early tissue damage regulating inflammation and apoptosis and eliciting neuroprotective pathways thereby maintaining patency of neurovascular units.

## Data Availability

Data sharing is not applicable to this article as no new data were created or analyzed in this study.

## References

[1] Adamou A , Beltsios ET , Bania A , Gkana A , Kastrup A , Chatziioannou A , et al. Artificial intelligence‐driven ASPECTS for the detection of early stroke changes in non‐contrast CT: a systematic review and meta‐analysis. J Neurointerv Surg. 2022. Online ahead of print. 10.1136/jnis-2022-019447 36522179

[2] Puleo MG , Miceli S , Di Chiara T , Pizzo GM , Della Corte V , Simonetta I , et al. Molecular mechanisms of inflammasome in ischemic stroke pathogenesis. Pharmaceuticals (Basel). 2022;15(10):1168. 36297283 10.3390/ph15101168 PMC9612213

[3] Peng L , Hu G , Yao Q , Wu J , He Z , Law BYK , et al. Microglia autophagy in ischemic stroke: a double‐edged sword. Front Immunol. 2022;13:1013311. 36466850 10.3389/fimmu.2022.1013311 PMC9708732

[4] Slevin M , Garcia‐Lara E , Capitanescu B , Sanfeliu C , Zeinolabediny Y , AlBaradie R , et al. Monomeric C‐reactive protein aggravates secondary degeneration after intracerebral haemorrhagic stroke and may function as a sensor for systemic inflammation. J Clin Med. 2020;9(9):3053. 32971821 10.3390/jcm9093053 PMC7563733

[5] Zhang Z , Liu Y , Liu Y and Wang Y . (2022). Friends or foes: the mononuclear phagocyte system in ischaemic stroke. 10.1111/bpa.13151 PMC10041168 36755470

[6] Monsour M , Croci DM , Agazzi S , Borlongan CV . Contemplating IL‐6, a double‐edged sword cytokine: which side to use for stroke pathology? CNS Neurosci Ther. 2022. Online ahead of print. 10.1111/cns.14041 PMC9873516 36478506

[7] Han‐Zu F , Yang C , Qiao L , Wang D , Li H , Yang L , et al. LAMO‐2A ablation in hippocampal CA1 astrocytes confers cerebroprotection and ameliorates neuronal injury after global brain injury. Brain Pathol. 2022;2022:e13114. 10.1111/bpa.13114 PMC10041161 36059143

[8] Gorantla NV , Chinnathambi S . Autophagic pathways to clear the tau aggregates in Alzheimer's disease. Cell Mol Neurobiol. 2021;41(6):1175–81. 32529542 10.1007/s10571-020-00897-0 PMC11448645

[9] Jiang L , Zhou L , Yong W , Cui J , Geng W , Chen H , et al. A deep learning‐based model for prediction of hemorrhagic transformation after stroke. Brain Pathol. 2021;e13023. Online ahead of print. 34608705 10.1111/bpa.13023 PMC10041160

[10] Zheng T , Tang W , Gup R , Xu WH . Studying the imaging features and infarction mechanism of vertebrobasilar dolichoectasia with high resolution MRI. BMC Neurol. 2017;17(1):216. 36718993 10.1111/bpa.13135 PMC10041158

